# Analysing the Anticancer Properties of Pterostilbene Through Absorption, Distribution, Metabolism, and Excretion (ADME) and Molecular Docking Studies

**DOI:** 10.7759/cureus.58425

**Published:** 2024-04-16

**Authors:** Monisha Prasad, Silambarasan Tamil Selvan, Rajeshkumar Shanmugam, Ramadurai Murugan, Mohammad Fareed

**Affiliations:** 1 Centre for Global Health Research, Saveetha Medical College and Hospital, Saveetha Institute of Medical and Technical Sciences, Chennai, IND; 2 Nanobiomedicine Lab, Centre for Global Health Research, Saveetha Medical College and Hospital, Saveetha Institute of Medical and Technical Sciences, Chennai, IND

**Keywords:** wnt signaling pathway, molecular docking, phytotherapy, pterostilbene (pts), liver cancer

## Abstract

Aim

The aim of this study is to examine the possible therapeutic effect of pterostilbene (PTS), a chemical present in grapes and blueberries, in the treatment of liver cancer by analysing its interactions with important proteins linked to the wingless/integrated (Wnt) signaling system.

Objective

Using computational techniques like molecular docking and absorption, distribution, metabolism, and excretion (ADME) studies, this research focuses on examining the pharmacokinetics and molecular interactions of PTS with proteins such as vimentin (Vim), glycogen synthase kinase 3 beta (GSK3-β), epithelial cadherin (E-cadherin), interleukin-6 (IL-6), interleukin-1 beta (IL-1β), c-Jun N-terminal kinase (JNK), and Wnt, all of which are connected to the Wnt signaling pathway in liver cancer.

Methods

The study includes the synthesis of proteins and ligands, ADME investigations for PTS, and AutoDock Vina molecular docking simulations to evaluate binding affinities and interactions. PTS is obtained from PubChem, while protein structures are obtained from the Protein Data Bank.

Results

Strong binding affinities between PTS and essential proteins in the Wnt signaling cascade are shown by molecular docking, which also highlights noteworthy hydrogen bonds, hydrophobic interactions, and electrostatic contacts. According to an ADME study, PTS has advantageous pharmacokinetic properties, such as moderate solubility, membrane permeability, and a minimal chance of drug interactions.

Conclusion

The extensive study highlights PTS's potential as a viable treatment option for liver cancer. The study promotes its investigation in cutting-edge liver cancer therapy approaches and urges more investigation into the molecular mechanisms, underpinning its anticancer properties. This paper sheds important light on the role of natural chemicals in cancer therapy and emphasizes the need for computational methods in drug discovery.

## Introduction

Liver cancer represents a significant global health challenge, characterized by its high incidence rates and limited treatment options. Particularly prevalent in developing nations, liver illnesses exacerbate the urgency of identifying effective therapies [1]. Traditional treatment modalities such as surgery, chemotherapy, and radiation often prove inadequate, especially as the disease progresses [2]. Consequently, there is an urgent need to explore novel therapeutic avenues to address this pressing health issue.

Central to the pathogenesis of liver cancer is the dysregulation of the wingless/integrated (Wnt) signaling pathway, a fundamental molecular cascade crucial for various cellular processes. Aberrant activation of the Wnt signaling pathway, primarily driven by dysregulation of β-catenin, serves as a key initiator and sustainer of hepatocellular carcinoma (HCC) [3]. This dysregulation occurs when Wnt ligands bind to cell surface receptors, leading to the inhibition of β-catenin degradation and subsequent accumulation in the nucleus, where it activates the expression of target genes implicated in carcinogenesis [4].

In liver cancer, mutations or dysregulation of key components of the Wnt pathway, such as β-catenin, adenomatous polyposis coli (APC), and Axin, results in the constitutive activation of Wnt signaling, thereby promoting tumor progression [5]. Additionally, crosstalk with other signaling pathways further amplifies the oncogenic potential of the Wnt pathway in liver cancer [6].

Therapeutic targeting of the Wnt pathway has emerged as a promising strategy for liver cancer treatment. Current efforts are focused on developing inhibitors that specifically target β-catenin and downstream effectors to effectively curb tumor growth. Furthermore, combination therapies that simultaneously target multiple pathways, including the Wnt pathway, hold promise for overcoming drug resistance and improving treatment efficacy [7].

Phytotherapy, which utilizes plant-derived substances, offers a promising avenue for liver cancer treatment [8]. Pterostilbene (PTS), a derivative of resveratrol commonly found in grapes and blueberries, has garnered attention for its potential medicinal applications. Its multifunctional properties, including its role in mediating disease resistance in plants, make it a subject of great interest [9].

A recent study conducted by Benlloch et al. [10] aimed to explore the therapeutic potential of PTS in liver cancer using computational techniques such as molecular docking and absorption, distribution, metabolism, and excretion (ADME) research. This study sought to elucidate the interactions between PTS and Wnt pathway proteins, as well as to investigate their pharmacokinetics. The insights gained from this study are crucial for guiding further experimental validations and gaining a deeper understanding of PTS's role in the treatment of liver cancer [11-13].

Furthermore, our recent publication delved into the complex relationships between PTS and liver cancer. Through the identification of significant differentially expressed genes (DEGs) in HCC and the elucidation of enriched pathways, this study underscored the potential of PTS as a therapeutic target in HCC by modulating essential signaling pathways [14].

Building upon these findings, we expanded our investigation to explore the molecular mechanisms underlying PTS's anticancer effects within the Wnt pathway. This endeavor is crucial for advancing PTS as a promising treatment option for liver cancer. By elucidating the intricate interplay between PTS and the Wnt signaling pathway, we aim to contribute to the development of innovative and effective therapeutic strategies for liver cancer management. Through comprehensive research efforts, including computational studies and experimental validations, we strive to harness the therapeutic potential of PTS and advance our understanding of its role in liver cancer therapy.

## Materials and methods

Protein retrieval and active site prediction

The Research Collaboratory for Structural Bioinformatics (RCSB) Protein Data Bank (PDB) was used to retrieve the protein structures of Wnt, vimentin (Vim), glycogen synthase kinase 3 beta (GSK-3β), epithelial cadherin (E-cadherin), interleukin-6 (IL-6), interleukin-1 beta (IL-1β), and c-Jun N-terminal kinase (JNK). The corresponding PDB IDs were 6AHY (Wnt), 8RVE (Vim), 5KPK (GSK-3β), 2O72 (E-cadherin), 1ALU (IL-6), 91LB (IL-1β), and 4YR8 (JNK). The "build/check/repair model" module was then used to carefully validate and repair these PDB files. This process involved introducing hydrogen molecules for complete structural integrity, modifying water molecules and atoms, and adding missing side chains through small regularization. AutoDockTools (ADT) (version 1.5.6) was used to process the corrected PDB files further in order to create .pdbqt files, which are necessary for the docking simulations that arrive. In order to preserve solely polar hydrogen atoms, non-standard residues and water molecules have to be removed during processing. Additionally, to assure accurate modeling of molecular interactions, the Kollman charges for the protein atoms were estimated using ADT.

Ligand preparation

The structure of our ligand, PTS, was retrieved from the PubChem database (PubChem ID: 5281727). To improve the geometry of the construction, Avogadro 4.2.1 software was used for geometric optimization. The .pdbqt file that was ready for AutoDock4 docking was based on this optimized structure. In order to make sure that the compound's geometry would work with the docking simulations, partial charges were applied. For simple integration into the docking studies, the resulting .mol2 file was subsequently transformed using ADT into a .pdbqt format.

Molecular docking

AutoDock4's (version 4.2.6) GALS search engine was used for docking studies. To identify the region of interest, which includes the entire catalytic binding site of every protein, a grid with 0.625 Å spacing was used. After 100 repetitions of the docking simulations, a cluster analysis with a root mean square (RMS) tolerance of 2.0 was performed to find the best binding postures. Cluster poses that demonstrated interactions that were energetically advantageous were assessed further in Biovia Discovery Studio in order to obtain a better understanding of ligand-protein interactions and possible binding affinities.

ADME studies for PTS

A thorough in silico pharmacokinetic evaluation of PTS was conducted using the Swiss ADME online software, aiming to provide insights into its interactions with biological systems and potential effects on human health. Adhering to the Lipinski rule of five, the analysis focused on key parameters (molecular weight, topological polar surface area (TPSA), rotatable bonds, hydrogen donor and acceptor atoms, miLog P) to assess absorption, distribution, metabolization, and excretion, as well as drug-like qualities [15].

Beyond pharmacokinetic characteristics, the computational analysis explored the compound's potential interactions with crucial biological components. Predictions were made regarding its effects on P-glycoproteins, cytochromes P450, and the blood-brain barrier (BBB), providing valuable information about safety and potential efficacy. This in silico pharmacokinetic study offered a predictive framework for understanding PTS's behavior in the human body, informing decisions about its therapeutic potential and guiding subsequent experimental trials [16].

## Results

Molecular docking

The results of our molecular docking investigation indicate that PTS has strong binding affinities with several important proteins. The complex relationships that PTS has with many target proteins are revealed by this study, highlighting the potential therapeutic benefits of PTS along several different biological pathways. The interaction between each protein and PTS is thoroughly examined in the sections that follow, providing insight into the distinct binding mechanisms and potential therapeutic uses of each protein (Table 1).

**Table 1:** Docking interactions of various proteins with ligands: scores, amino acid involvement, distances, and interaction types Wnt: wingless/integrated; Vim: vimentin; IL-6: interleukin-6; IL-1β: interleukin-1 beta; JNK: c-Jun N-terminal kinase; GSK-3β: glycogen synthase kinase 3 beta; E-cadherin: epithelial cadherin

S. no.	Protein name	Docking score	Amino acid interaction	Distance	Interaction type	Interaction bond
1	Wnt	-7.7	ASN103	3.02845	Hydrogen bond	Conventional hydrogen bond
			ARG313	2.98679	Hydrogen bond	Conventional hydrogen bond
			ASN99	3.52201	Hydrogen bond	Carbon-hydrogen bond
			ARG313	4.56419	Electrostatic	Pi-cation
			ALA314	4.26438	Hydrophobic	Alkyl
			ARG313	4.21772	Hydrophobic	Alkyl
			ARG98	5.10021	Hydrophobic	Pi-alkyl
			LYS94	4.29412	Hydrophobic	Pi-alkyl
			ARG98	5.33725	Hydrophobic	Pi-alkyl
2	Vim	-6.9	TYR383	3.01941	Hydrogen bond	Conventional hydrogen bond
			GLN343	2.91845	Hydrogen bond	Conventional hydrogen bond
			GLU382	3.59385	Electrostatic	Pi-anion
			PHE351	5.16578	Hydrophobic	Pi-Pi T-shaped
			ALA355	3.91078	Hydrophobic	Alkyl
			TYR383	5.01273	Hydrophobic	Pi-alkyl
			MET347	4.36032	Hydrophobic	Pi-alkyl
			VAL389	5.03979	Hydrophobic	Pi-alkyl
3	IL-6	-6.8	SER70	2.54215	Hydrogen bond	Conventional hydrogen bond
			SER73	2.97656	Hydrogen bond	Carbon-hydrogen bond
			PRO78	2.76726	Hydrogen bond	Carbon-hydrogen bond
			GLU74	3.55136	Hydrogen bond	Carbon-hydrogen bond
			LYS66	4.94718	Hydrophobic	Alkyl
			ALA69	4.80017	Hydrophobic	Pi-alkyl
			ILE64	4.81585	Hydrophobic	Pi-alkyl
			VAL76	4.51025	Hydrophobic	Pi-alkyl
4	IL-1β	-7.3	LEU30	1.95165	Hydrogen bond	Conventional hydrogen bond
			LEU80	2.0934	Hydrogen bond	Conventional hydrogen bond
			GLU75	2.39737	Hydrogen bond	Conventional hydrogen bond
			VAL131	3.40029	Hydrogen bond	Carbon-hydrogen bond
			ASN28	3.68677	Hydrogen bond	Carbon-hydrogen bond
			LEU30	4.68451	Hydrophobic	Alkyl
			LEU78	4.62248	Hydrophobic	Alkyl
			VAL131	4.7378	Hydrophobic	Alkyl
			LEU80	4.98337	Hydrophobic	Alkyl
			ARG77	4.36184	Hydrophobic	Pi-alkyl
5	JNK	-7.3	A:GLN37:NE2 - :UNK0:O1	3.20453	Hydrogen bond	Conventional hydrogen bond
			A:ARG69:NH1 - :UNK0	3.38555	Electrostatic	Pi-cation
			A:VAL187:CG2 - :UNK0	3.61191	Hydrophobic	Pi-sigma
			A:TYR185 - :UNK0	5.27326	Hydrophobic	Pi-Pi T-shaped
			A:ALA36 - :UNK0:C18	4.47472	Hydrophobic	Alkyl
			:UNK0:C18 - A:VAL187	4.5206	Hydrophobic	Alkyl
			:UNK0:C19 - A:LEU172	5.19402	Hydrophobic	Alkyl
			:UNK0:C19 - A:MET182	4.62349	Hydrophobic	Alkyl
			A:TYR185 - :UNK0:C19	4.78061	Hydrophobic	Pi-alkyl
			:UNK0 - A:ALA36	4.81671	Hydrophobic	Pi-alkyl
			:UNK0 - A:LEU172	5.25578	Hydrophobic	Pi-alkyl
			:UNK0 - A:ALA36	5.04938	Hydrophobic	Pi-alkyl
6	GSK-3β	-7.4	VAL135	3.15393	Hydrogen bond	Conventional hydrogen bond
			ASP133	2.38281	Hydrogen bond	Conventional hydrogen bond
			TYR134	3.30711	Hydrogen bond	Carbon-hydrogen bond
			THR138	3.58451	Hydrogen bond	Carbon-hydrogen bond
			ASN186	3.47055	Hydrogen bond	Carbon-hydrogen bond
			ASP200	3.65622	Hydrogen bond	Carbon-hydrogen bond
			LEU188	3.78738	Hydrophobic	Pi-sigma
			LEU188	3.77084	Hydrophobic	Pi-sigma
			ARG141	4.90721	Hydrophobic	Alkyl
			TYR140	4.94704	Hydrophobic	Pi-alkyl
			VAL70	5.29881	Hydrophobic	Pi-alkyl
			ALA83	4.35883	Hydrophobic	Pi-alkyl
			CYS199	5.30912	Hydrophobic	Pi-alkyl
7	E-cadherin	-7.1	ASN20	2.25315	Hydrogen bond	Conventional hydrogen bond
			SER8	3.92844	Hydrogen bond	Pi-donor hydrogen bond
			LEU21	4.98262	Hydrophobic	Alkyl
			PRO5	5.22066	Hydrophobic	Alkyl
			ILE7	4.35991	Hydrophobic	Alkyl
			VAL22	4.26982	Hydrophobic	Alkyl
			ILE7	4.86732	Hydrophobic	Pi-alkyl
			PRO6	5.22602	Hydrophobic	Pi-alkyl

Exposing PTS's binding with Wnt protein

**Figure 1:**
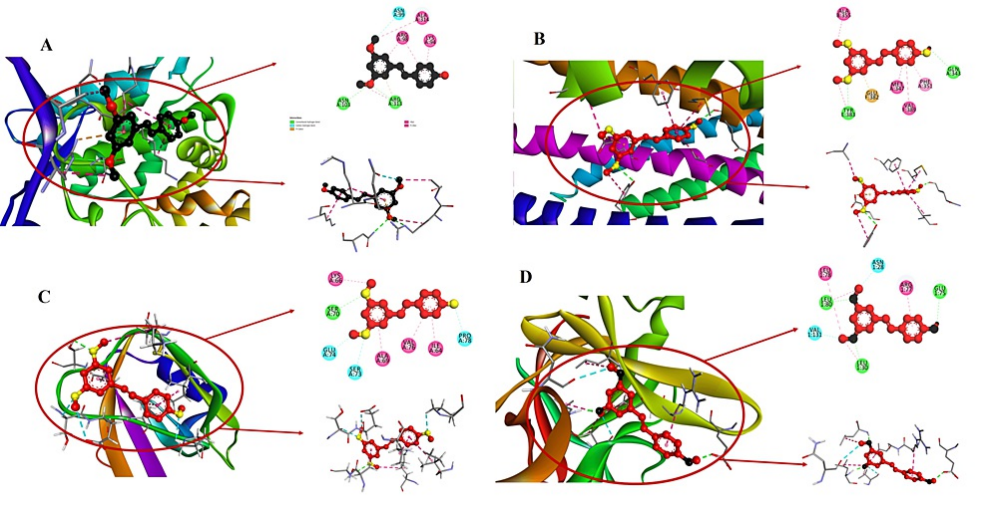
Investigating molecular docking interactions: PTS binding affinities with Wnt, Vim, IL-6, and IL-1β proteins Molecular docking of PTS with key proteins like (A) Wnt, (B) Vim, (C) IL-6, and (D) IL-1β, revealing strong affinities and specific amino acid interactions. AutoDock version 1.5.6 was employed for the study, and the figure was generated using visualization software PTS: pterostilbene; Wnt: wingless/integrated; Vim: vimentin; IL-6: interleukin-6; IL-1β: interleukin-1 beta

PTS has a significant binding affinity as seen by its robust docking score of -7.7 with Wnt (Figure 1A). ASN103 and ARG313 are two important amino acids that participate in hydrogen bonding in the interaction profile, with distances of 3.02845 and 2.98679, respectively. Furthermore, the binding interface's adaptability and intricacy are highlighted by hydrophobic interactions with ALA314 and ARG313 as well as electrostatic contacts and Pi-cation bonds with ARG313.

Exposing PTS's binding with Vim protein

A significant score of -6.9 is found in the docking analysis for the interaction between PTS and Vim (Figure 1B). The typical hydrogen bonding distances for amino acids TYR383 and GLN343 are 3.01941 and 2.91845, respectively. A thorough understanding of the complex binding modes is provided by electrostatic interactions with GLU382 and a variety of hydrophobic interactions, such as Pi-Pi T-shaped interactions with PHE351.

The binding landscape of PTS with IL-6

PTS interacts with IL-6 with a significant docking score of -6.8 (Figure 1C). Important amino acids have conventional hydrogen bonding interactions at distances of 2.54215 and 2.97656, respectively, with SER70 and SER73. The stable binding is facilitated by Pi-alkyl interactions with ALA69 and hydrophobic interactions with LYS66, suggesting possible therapeutic relevance in IL-6 regulation.

The binding landscape of PTS with IL-1β

A strong binding affinity of -7.3 between PTS and IL-1β is revealed by molecular docking (Figure 1D). Distinguished amino acids, such as LEU30 and LEU80, establish standard hydrogen bonds at 1.95165 and 2.0934, in that order. ARG77's Pi-alkyl interactions in conjunction with LEU30 and LEU78's hydrophobic contacts point to a flexible way of interaction with IL-1β.

Exposing PTS's binding with JNK protein

**Figure 2:**
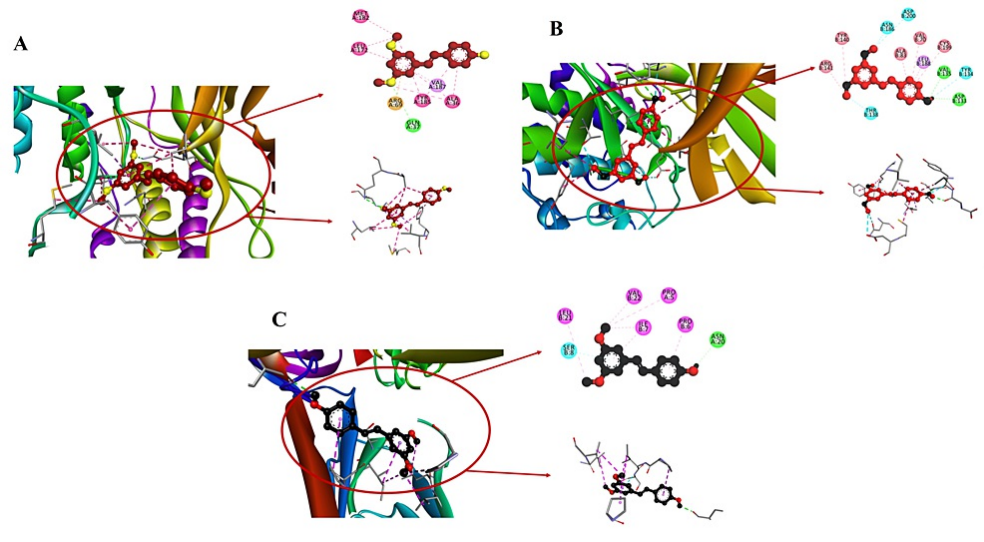
Deciphering PTS molecular docking interactions with JNK, GSK3-β, and E-cadherin: revealing significant binding affinities and amino acid interactions Molecular docking of PTS with (A) JNK, (B) GSK-3β, and (C) E-cadherin, employing AutoDock version 1.5.6. The analysis demonstrates notable binding affinities, highlighting essential amino acid interactions, including hydrogen bonds and hydrophobic contacts. Visualization software was used for figure generation PTS: pterostilbene; JNK: c-Jun N-terminal kinase; GSK-3β: glycogen synthase kinase 3 beta; E-cadherin: epithelial cadherin

A docking score of -7.3 characterizes the interaction between PTS and JNK (Figure 2A). Hydrogen bonds with A: GLN37:NE2 and electrostatic interactions with A: ARG69:NH1 are important interactions. Pi-alkyl and hydrophobic interactions with different amino acids shed light on the different binding mechanisms and raise the possibility of therapeutic applications in the control of the JNK pathway.

Exposing PTS's binding with GSK-3β protein

PTS interacts with GSK-3β with a significant docking score of -7.4 (Figure 2B). Conventional hydrogen bonds are formed by essential amino acids such as ASP133 and VAL135 at distances of 2.38281 and 3.15393, respectively. Pi-sigma with LEU188 and Pi-alkyl with TYR140 are two examples of hydrophobic interactions that highlight the complex binding kinetics.

The binding landscape of PTS with E-cadherin

A significant docking score of -7.1 indicates the nature of the interaction between PTS and E-cadherin (Figure 2C). Pi-alkyl interactions with PRO6 and hydrophobic contacts with LEU21 contribute to the stable binding interface, while amino acids ASN20 and SER8 engage in traditional hydrogen bonds. This emphasizes the broad therapeutic possibilities of PTS by indicating its capacity to impact E-cadherin-related pathways.

ADME studies for PTS

**Figure 3:**
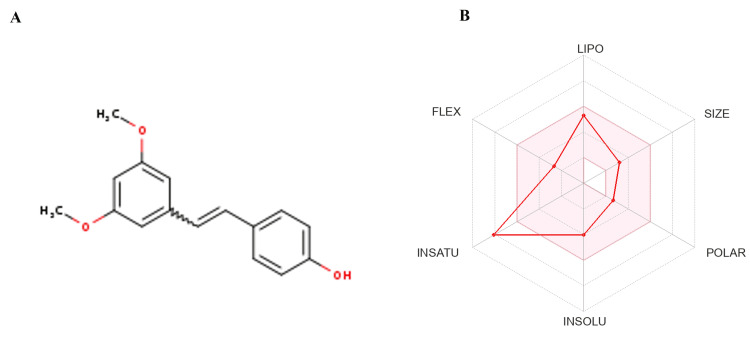
Physiochemical insights into PTS: 2D structure (A) and Lipinski violations (B) Physiochemical properties of PTS. The (A) 2D structure of PTS is presented on the right, while (B) Lipinski violations are highlighted in red on the left. The image was extracted from the SwissADME website tool PTS: pterostilbene

ADME tests were conducted to assess the pharmacokinetic features of PTS, which has a molecular weight of 256.3 g/mol and a formula of C16H16O3 (Table 2). According to its Log P values (XLOGP3: 3.78, WLOGP: 3.36, MLOGP: 2.76, Consensus Log P: 3.61), the chemical showed moderate solubility, suggesting favourable lipophilicity (Figure 3A). PTS has the potential to penetrate membranes while having a low water solubility (Log S: -4.01). According to Estimated SOLubility (ESOL), it was categorised as somewhat soluble. Its potential for systemic and central nervous system impacts is highlighted by predictions that indicate gastrointestinal absorption and BBB permeability. The main cytochrome P450 enzymes (CYP1A2, CYP2C19, CYP2C9, CYP2D6, CYP3A4) did not interact significantly with PTS, suggesting a decreased risk of drug-drug interactions. Good oral bioavailability and ideal drug-likeness are suggested by following the guidelines set forth by Lipinski, Veber, and Ghose. PTS has a 2.29 synthetic accessibility score, which indicates that its synthesis is reasonably accessible (Figure 3B). These results together point to favourable pharmacokinetic properties for the compound's prospective use as a bioactive agent.

**Table 2:** ADME properties associated with PTS SMILES: simplified molecular-input line-entry system; TPSA: topological polar surface area; BBB: blood-brain barrier; GI: gastrointestinal; ADME: absorption, distribution, metabolism, and excretion: PTS: pterostilbene

Physiochemical properties	
Mol wt (g/mol)	256.3
Formula	C16H16O3
Canonical SMILES	COC1=CC(=CC(=C1)C=CC2=CC=C(C=C2)O)OC
TPSA	38.69
BBB permeant	Yes
GI absorption	High
Lipinski violations	Yes; 0 violation
Bioavailability score	0.55
Synthetic accessibility	2.29
Water solubility	Moderately soluble

## Discussion

The fact that liver cancer is still a major hazard to world health highlights the critical need for novel therapeutic approaches. Examining natural substances has become a viable approach, especially when it comes to treating liver cancer. Because of its unique chemical composition and biological characteristics, PTS, a resveratrol derivative that can be obtained from a variety of dietary sources, stands out as a strong contender [17,18]. Through the use of an in silico approach, our research previously showed the promise of PTS as a therapeutic agent for liver cancer, specifically HCC. This study examined the effects of PTS on DEGs found in HCC. The results demonstrated the complex relationships between PTS and liver cancer, highlighting the compound's promise as a treatment target. This preliminary finding emphasizes the further study needed to develop targeted HCC treatments.

This research employs a combination of molecular docking and ADME experiments to investigate the anticancer properties of PTS. By integrating computational methodologies, our aim is to gain a comprehensive understanding of the molecular interactions and key pharmacokinetic processes that govern PTS's action within the human body.

The development of HCC is regarded as a multistage process in which multiple genetic alterations are necessary. The Wnt pathway is a signaling mechanism that is frequently activated in HCC, especially the canonical Wnt pathway. Moreover, two main non-canonical pathways are also involved in the regulation of hepatocarcinogenesis. Interestingly, the non-canonical Wnt pathway could antagonize the canonical Wnt pathway in HCC. Crosstalk between other signaling pathways and the Wnt pathway has also been shown to promote tumorigenesis [3]. Conversely, the remarkable anticancer properties of PTS have been documented across various tumor types, including lung, colon, breast, and cervical cancers. PTS has demonstrated significant efficacy in suppressing cancer progression and metastasis by modulating both apoptosis-dependent and apoptosis-independent signaling pathways [19]. However, its potential as an anticancer agent through the regulation of the Wnt signaling pathway remains unexplored. This study aims to elucidate the mechanisms of action of PTS as an anticancer agent, shedding light on its pharmacological profile by investigating its effects on proteins involved in the Wnt signaling pathway in HCC.

The extensive molecular docking results provide compelling insights into PTS's potent anti-inflammatory effects, particularly within the Wnt signaling pathway. Strong binding affinities across proteins associated with the Wnt pathway suggest PTS's potential to modulate this critical pathway implicated in inflammation and tumor growth, notably in HCC [20]. Given the aberrant activation of the Wnt pathway in liver cancer, targeting it is pivotal for therapeutic interventions. PTS's ability to interact strongly with proteins within the Wnt pathway implies its potential in mitigating inflammatory responses associated with cancer progression.

Furthermore, the molecular docking analysis reveals the exceptional binding affinity of PTS with crucial proteins implicated in various biological pathways. Particularly noteworthy are its robust interactions with proteins such as Vim, IL-6, and GSK-3β, indicative of its broad therapeutic potential across multiple pathways [21]. Vim, an intermediate filament protein, plays a pivotal role in maintaining cell shape and integrity. Its involvement in cancer progression, metastasis, and drug resistance makes it an attractive target for anticancer therapies [22]. PTS's strong interaction with Vim suggests its potential to disrupt these processes, thereby inhibiting tumor growth and metastasis. IL-6 is a pro-inflammatory cytokine implicated in various aspects of cancer development, including proliferation, survival, and angiogenesis. By targeting IL-6 signaling, PTS may suppress inflammation-driven tumorigenesis and inhibit cancer cell proliferation and survival [23].

GSK-3β is a key regulator of diverse cellular processes, including cell proliferation, apoptosis, and differentiation. Dysregulation of GSK-3β signaling is implicated in various diseases, including cancer [24]. PTS's strong interaction with GSK-3β suggests its potential to modulate these pathways, thereby exerting anticancer effects. Furthermore, insights from PTS research extend to its molecular-level effects on eukaryotic cells [19]. Studies using model organisms reveal significant alterations in cellular pathways, emphasizing PTS's anti-inflammatory properties and its potential implications in cancer. Notably, PTS demonstrates promising antitumor activity against breast cancer, including triple-negative breast cancer cells, highlighting its broad therapeutic potential across various cancer types [25].

Moreover, an essential step in understanding PTS's therapeutic efficacy is the assessment of its ADME properties. With favourable pharmacokinetic properties and adherence to drug-likeness guidelines, PTS emerges as a promising bioactive agent in therapeutic applications, including liver cancer treatment. In conclusion, this study sheds light on the relationships and effects of PTS on liver cancer, uncovering its potential as a therapeutic agent. Molecular docking analysis reveals strong binding affinities with important cancer-related proteins, while ADME experiments demonstrate positive pharmacokinetic properties. Together, these findings underscore PTS's potential as a therapeutic agent in liver cancer.

## Conclusions

The possible anticancer effects of PTS are explored in this paper, with a focus on liver cancer. Molecular docking analyses and ADME tests are used to thoroughly evaluate this natural compound's medicinal value. The research highlights the critical role of computational approaches, specifically molecular docking, in the early stages of drug design, significantly reducing the costs associated with drug production. The comprehensive exploration of PTS through computational approaches and pharmacokinetic assessments opens avenues for further research and potential clinical applications. The findings support more research into the molecular basis of PTS's anticancer properties and its possible application in novel and powerful liver cancer therapies. With every aspect considered, this research contributes to the expanding reservoir of information about natural substances and their uses in cancer treatment.
